# Proguanil synergistically sensitizes ovarian cancer cells to olaparib by increasing DNA damage and inducing apoptosis

**DOI:** 10.7150/ijms.67027

**Published:** 2022-01-01

**Authors:** Yan Wu, Tianyu Wu, Xin Hu, Simeng Xu, Di Xiao, Jingtao Wu, Xinjian Yan, Xiaoping Yang, Gaofeng Li

**Affiliations:** 1 Department of Oncology, Zhuzhou Hospital Affiliated to Xiangya School of Medicine, Central South University, Zhuzhou 412000, Hunan, China.; 2 Key Laboratory of Study and Discovery of Small Targeted Molecules of Hunan Province, Department of Pharmacy School of Medicine, Hunan Normal University, Changsha 410013, Hunan, China.

**Keywords:** Proguanil, olaparib, ovarian cancer, combination therapy

## Abstract

Ovarian cancer is the second leading cause of cancer-related deaths in women, with low five-year survival rates. Therefore, it is essential to seek new treatment options. Olaparib, a PARP inhibitor, has benefited many ovarian cancer patients, but olaparib is much less effective as a single agent in 50% of patients with high grade severe tumors. Proguanil, which was originally developed as an anti-malarial drug, has gained attention due to its anti-tumor effects. Here, we evaluated the anti-tumor effect of the combination of olaparib and proguanil on ovarian cancer cells, aimed to develop a potential medical option for treating ovarian cancer patients. We examined the effect on proliferation by MTT and colony formation assays, while cell migration was measured by the transwell assay. The effect on apoptosis was measured by flow cytometry and AO/EB staining assays. Western blotting was used to detect protein expression levels in cells treated with olaparib and/or proguanil. In addition, the synergistic effect of these two drugs is calculated by CompuSyn software. The combination of olaparib and proguanil significantly increased growth suppression and apoptosis in ovarian cancer cells, compared to either single agent alone. Furthermore, results showed that the combination of olaparib and proguanil synergistically increased olaparib-induced apoptosis and DNA damage and reduced the efficiency of DNA homologous recombination repair. Our findings indicate that combination of olaparib with proguanil will be a novel potential administration route for treating ovarian cancer patients.

## Introduction

Ovarian cancer is a gynecological tumor with a high mortality rate, second only to breast cancer in gynecological tumors. Currently, there are nearly 13770 deaths and 21410 new cases of ovarian cancer in the United States every year 1. The main treatment methods are surgical resection and combination with various chemotherapies, but the recurrence of patients and drug resistance make the treatment prognosis poor. The five-year survival rate of advanced ovarian cancer has not been improved significantly for many years 2. Therefore, the development of new treatment is very urgent.

Olaparib became the first PARP inhibitor approved by FDA for treating BRCA1/2 mutated ovarian cancer patients who are receiving three or more chemotherapy regimens in 2014. The mechanism in the treatment of ovarian cancer is mainly through inhibiting homologous recombination repair, increasing DNA damage, realizing synthetic lethal and killing tumor cells 3. Although olaparib has achieved good clinical response, only about 50% of ovarian cancers are homologous recombination (HR) deficient, which makes PARP inhibitors ineffective to considerable amounts of ovarian cancer patients 4. Developing strategies to extend the use of PARP inhibitor to HR-proficient oncology is a key clinical challenge. One strategy is to develop rational combinations of PARPI with other drugs.

Proguanil, a member of the biguanide family, was originally developed as an anti-malarial drug 5. Interestingly, we noticed that some studies reported that proguanil may exert anticancer effects by reducing tumor hypoxia, inducing mitochondrial dysfunction and oxidative stress, and causing DNA damage 6. Moreover, compared with other biguanide drugs, proguanil continued to have the higher inhibitory effect on colon and bladder cancer cell growth 7. In other hand, the ability of proguanil to sensitize tumor cells to small molecular targeted drugs has not been explored and this area is worthy of deep exploration. Herein, we are trying to investigate the anti-tumor effects of proguanil in combination with olaparib on ovarian cancer cells, which may provide a new option for ovarian cancer treatment.

## Materials and Methods

### Reagents

Olaparib (AZD2281, Ku-0059436) was obtained from Targetmol (Shanghai, China). Proguanil was obtained from Selleck Chemicals (Shanghai, China). Acridine orange/ethidium bromide (AO/EB) staining was obtained from Kehang Biotechnology (Changsha, China), and the FITC Annexin V Apoptosis Detection kit was from BD Pharmingen (NJ, USA). Rabbit anti-human antibodies specific for the following proteins were obtained from Cell Signaling (MA, USA): Phospho-Histone H2A.X (Ser139) and β-actin. Phospho-ATM (Ser1987) was obtained from Abcam (MA, USA). BRCA1 (sc-6954) was obtained from Santa Cruz Biotechnology (Texas, USA). Bcl-2 polyclonal antibody and Bax polyclonal antibody were obtained from Bioworld Technology (MN, USA) and Biyotime (Shanghai, China) respectively.

### Cell culture

Human ovarian cancer SKOV3 and OVCAR3 cell lines provided by Xiangya Hospital Affiliated to Central South University (Changsha, Hunan, China) were cultured in RPMI-1640 (HyClone) medium supplemented with 10% fetal bovine serum (Hyclone) and 1% penicillin-streptomycin (Hyclone) at 37 °C in a humidified atmosphere with 5% CO_2_.

### MTT assay

MTT assay was used to detect the viability of ovarian cancer cells. Cells were added to 96 well plates (6×10^3^ cells/well) for 24 h, then olaparib and/or proguanil of different concentrations were treated for 72 h. Then 2 mg/mL MTT tetrazolium salt (Sigma) (50 µL) was added to each well for 5 hours, then 150 µL of DMSO (Sigma) was added to each well. The absorbance of each well was measured at 490nm using a microplate reader (BioTek, Synergy HTX, Vermont, USA).

### Calculation of Combination index (CI)

On the basis of the results of the MTT assay, CI values were calculated using CompuSyn software according to the Chou-Talay method 8. A CI level of <0.9, CI=0.9-1.1 and CI>1.1 indicates synergistic, additive and antagonistic effects of the two drug combinations, respectively 9.

### Colony formation assay

Colony formation was used to monitor the proliferation of ovarian cancer cells. Cells were placed in a 24-well plate at 5×10^3^/well and then treated with a series of olaparib and/or proguanil doses for 24 h and collected after 5-7 days. Next, the culture medium is discarded. Cells were carefully washed twice with PBS and fixed with 10% formalin for 2 h. Add 0.1% crystal violet solution to the dye and treat for 2 hours. The absorbance at 550 nm was measured by a microplate reader (BioTek, Synergy HTX, Vermont, USA).

### Transwell migration assay

Transwell assay was employed to evaluate the migration of ovarian cancer cells. 5×10^4^ cells were placed in 200 µl serum-free medium and added into the upper chamber, and the medium containing 10% fetal bovine serum was added into the lower chamber. Then appropriate olaparib and/or proguanil were added for 24 h, after which the cells in the upper compartment were removed, the remaining cells were fixed with 10% formaldehyde for 30 min, stained with 0.1% crystal for 2 h, and then imaged by microscope (Leica, DFC450C; Wetzlar, Germany). The number of migrated cells in three random fields was counted.

### Flow Cytometry Annexin V-FITC-PI Assay

Apoptotic cell death was assessed by Annexin V-FITC/PI double staining. In brief, ovarian cancer cells were placed in a 6-well plate at 5×10^5^ cells/well and treated with different doses of olaparib and proguanil for 24 h. Cells were collected and stained with Annexin V-FITC and PI (5 µl each) in a 300 µL volume and incubated in darkness for 20 min. The apoptotic cells were then evaluated by FACS Calibur flow cytometer (Becton, Dickinson and Company, New Jersey, USA).

### Microscopy Imaging and Acridine Orange-Ethidium Bromide Staining

Acridine Orange-Ethidium Bromide Staining was used to observe the cell apoptosis from the changes of cell morphology. Cells were added to 6 well plates (5×10^5^ cells/well) for 24 h, then olaparib and/or proguanil of different concentrations were added for 24 h. After that, cells were collected and cleaned with PBS once, and stained with AO/EB working solution for 5-15 min. The apoptotic cells were then observed and photographed via microscope (Leica, DFC450C; Wetzlar, Germany).

### Western Blotting

Whole cell lysates from each sample were loaded onto a 10% polyacrylamide gel for electrophoresis and transferred to a PVDF membrane. After the primary antibody was incubated overnight at 4 °C, it was washed with PBS/0.1% Tween-20, and then incubated at room temperature with the secondary antibody for 1 hour. The protein was visualized using Clarity Western ECL Substrate (Bio-Rad, California, USA) and imprinted immediately on the Chemidoc system (Tanon 4600, Shanghai, China). Protein expression was quantified using Image J.

### Statistical analyses

Data are expressed as ± standard deviation. Two independent groups were compared using two-tailed t-tests and two-way ANOVAs. All statistical analyses were performed within 95% confidence intervals and the corresponding P-values were reported (*P<0.05, **P<0.01, ***P<0.001). Statistical analysis was performed using GraphPad Prism 6 and SPSS 25.0.

## Results

### Cytotoxicity of the combination of olaparib and proguanil on ovarian cancer cells

**Figure 1:**
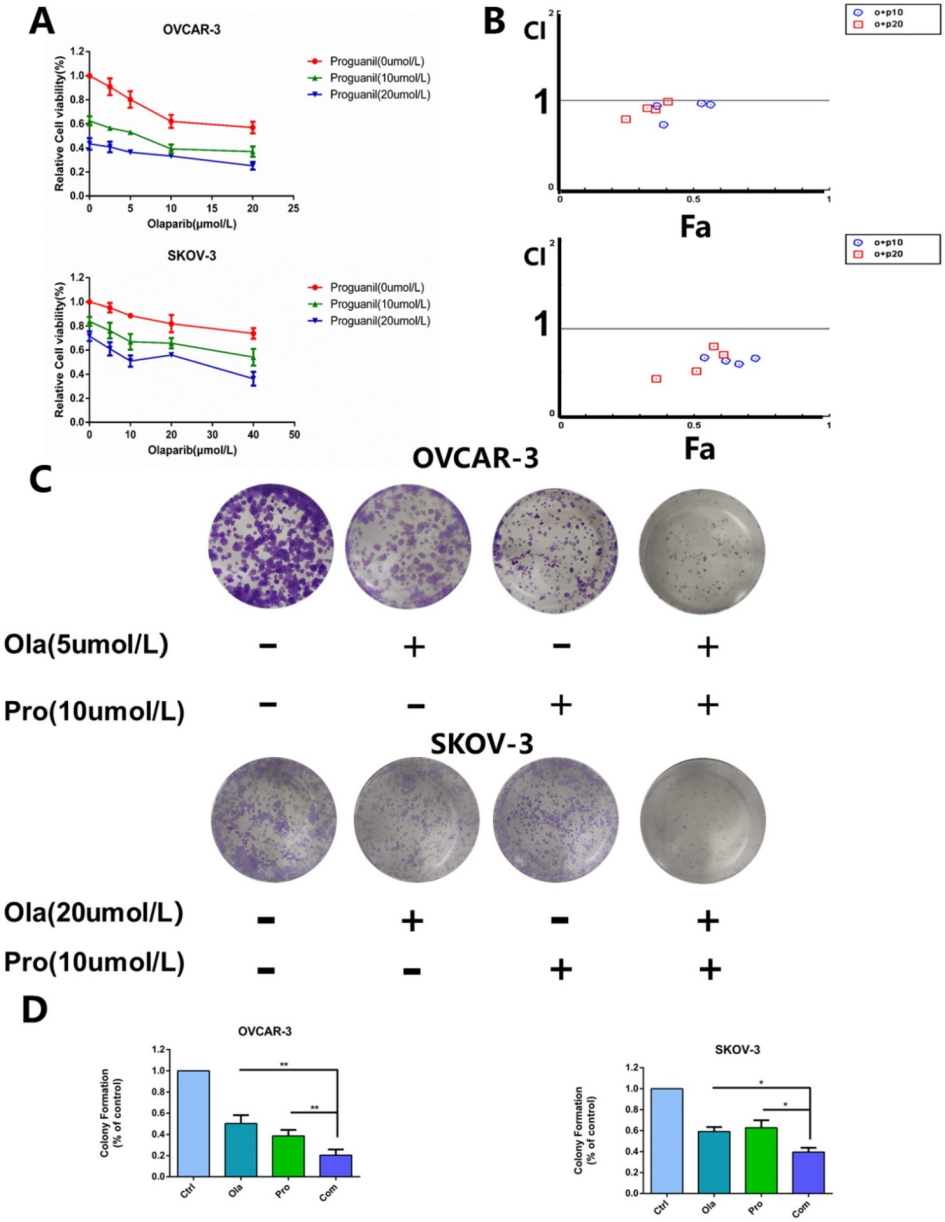
** Effects of olaparib combined with proguanil on proliferation and colony-forming activity of OVCAR-3 and SKOV-3 cells.** (A) Olaparib and proguanil synergistically inhibited cell proliferation of OVCAR-3 and SKOV-3 cells, which was measured at 72h post-treatment with olaparib and/or proguanil. (B) Synergy between two drugs is evaluated by the combination index (CI), with CI=0.9-1.1, CI>1.1 and CI<0.9 indicating additive, antagonistic and synergistic effects respectively. In the majority of cases, CI values were below 0.9, indicating a synergistic effect of two drugs. (C) Evaluation of the ability of olaparib and proguanil to suppress colony formation. Cells were incubated with olaparib and/or proguanil for 5-7 days and then photographed. (D) The colony-formation ability of each group was shown in bar chart and described as mean±s.d of three independent experiments. (*P < 0.05, **P < 0.01,n=3)

In this study, we evaluated the antitumor effect of the combination of olaparib and proguanil on human ovarian cancer cell lines, including OVCAR-3 and SKOV-3. Cells were treated with a serial concentration of olaparib, proguanil or both for 72 hours, and the survival rate of each treatment group was detected using MTT assay. We found different drug sensitivities in these two types of cells, which provided a reference for the selection of concentrations in our subsequent series of experiments. Dose-dependent inhibition of proliferation of both cell types by both drugs after 72 hours was witnessed, with estimated IC_50_ values of 21.7 umol/L (OVCAR-3) and 80.9 umol/L (SKOV-3) for olaparib, and 22.4 umol/L (OVCAR-3) and 45.7 umol/L (SKOV-3) for proguanil. Based on this finding, different drug concentrations were used for the two cell lines in subsequent experiments. The combination of the two drugs had an enhanced cell growth inhibitory effect, compared to each drug alone (Fig. 1A). The combination index of the two drugs was calculated by using CompuSyn software to determine whether the two drugs have synergistic effects. The level of CI for OVCAR-3 ranged from 0.726 to 0.991 and for SKOV-3 varied from 0.433 to 0.8, shown in Fig. 1B. Most CI values for OVCAR-3 and all CI values for SKOV-3 were less than 0.9, demonstrating a synergistic effect of the two drugs. A small minority of CI values for OVCAR-3 fell within 0.9 and 1, which represented a simple additive effect.

Next, we assessed whether the combination of olaparib and proguanil induced long-term synergistic effects in anti-tumor activity by colony formation. This assay showed that the combination of olaparib and proguanil was effective in inhibiting ovarian cancer cell colony formation (Fig. 1C-D). Taken together, the combination of olaparib and proguanil showed more potent antitumor effects against two human ovarian cancer cell lines.

### Olaparib and proguanil inhibit the migration of ovarian cancer cells

**Figure 2:**
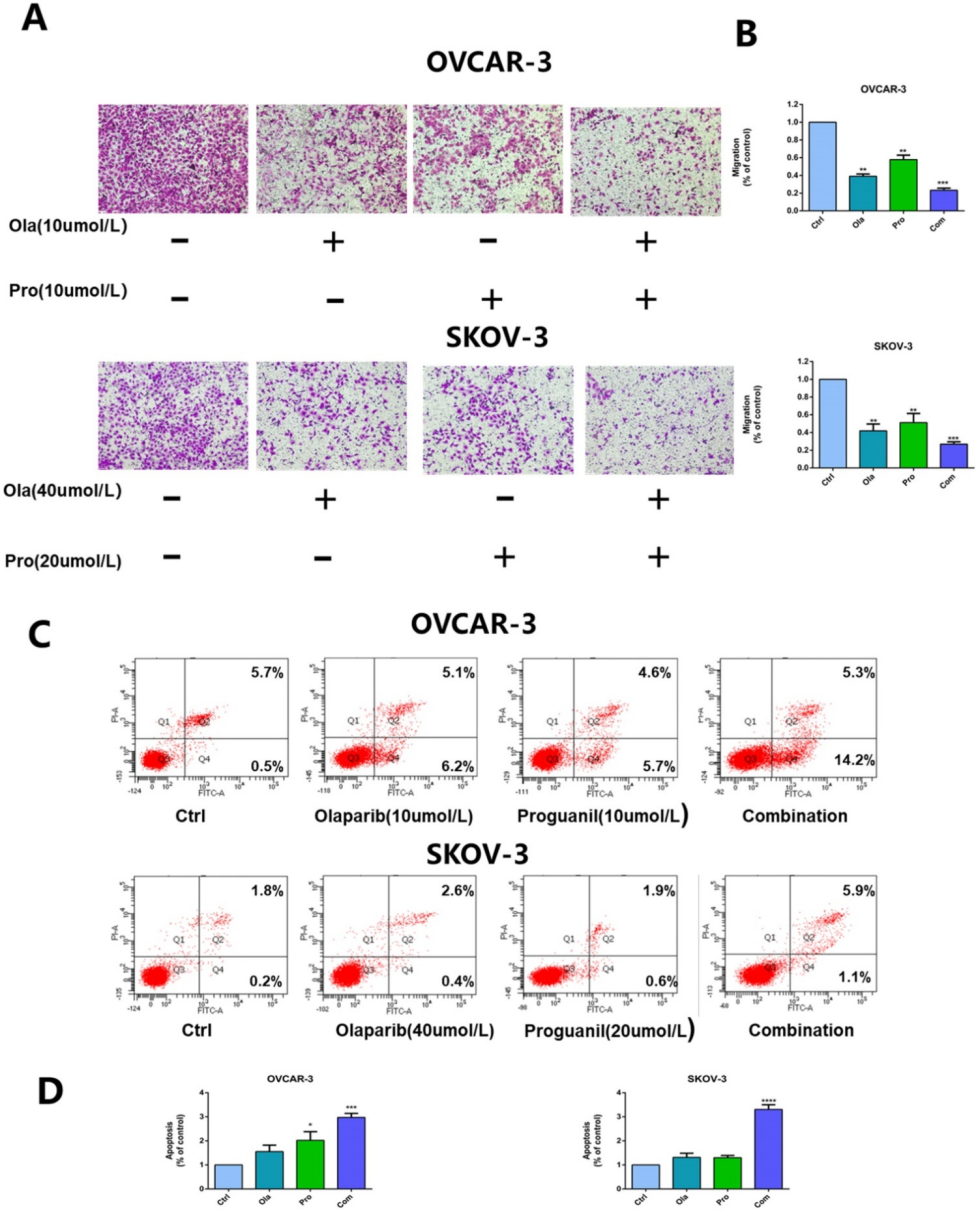
** Effects of olaparib combined with proguanil on migration and apoptosis of OVCAR-3 and SKOV-3 cells.** (A) Combination of olaparib and proguanil treatment suppressed the migratory activity of OVCAR-3 and SKOV-3 cells in n a transwell assay. (B) Quantification results were presented. (C) Representative flow cytometry plots corresponding to cells stained with propidium iodide (y-axis) and Annexin V-FITC (x-axis). (D)Quantification results were presented. (*P < 0.05, **P < 0.01, ***P < 0.001, n = 3).

Then**,** we used the transwell assay to assess the ability of olaparib and proguanil on inhibiting the migration of OVCAR-3 cells and SKOV-3, and the results showed that the combination of these two drugs was able to inhibit this migratory activity, much stronger than either drug alone (Fig. 2A-B).

### The combination of olaparib and proguanil results in enhanced apoptosis in ovarian cancer cells

To further evaluate the synergistic antitumor effects of the two drugs, we also monitored the apoptosis of OVCAR-3 cells and SKOV-3 cells after 24 hours drug treatment. Flow cytometry was used to detect cell apoptosis with Annexin V-FITC/PI double staining solution and the results showed that the combination of the two drugs enhanced cell apoptosis in ovarian cancer cells, compared monotherapy (Fig. 2C-D).

**Figure 3:**
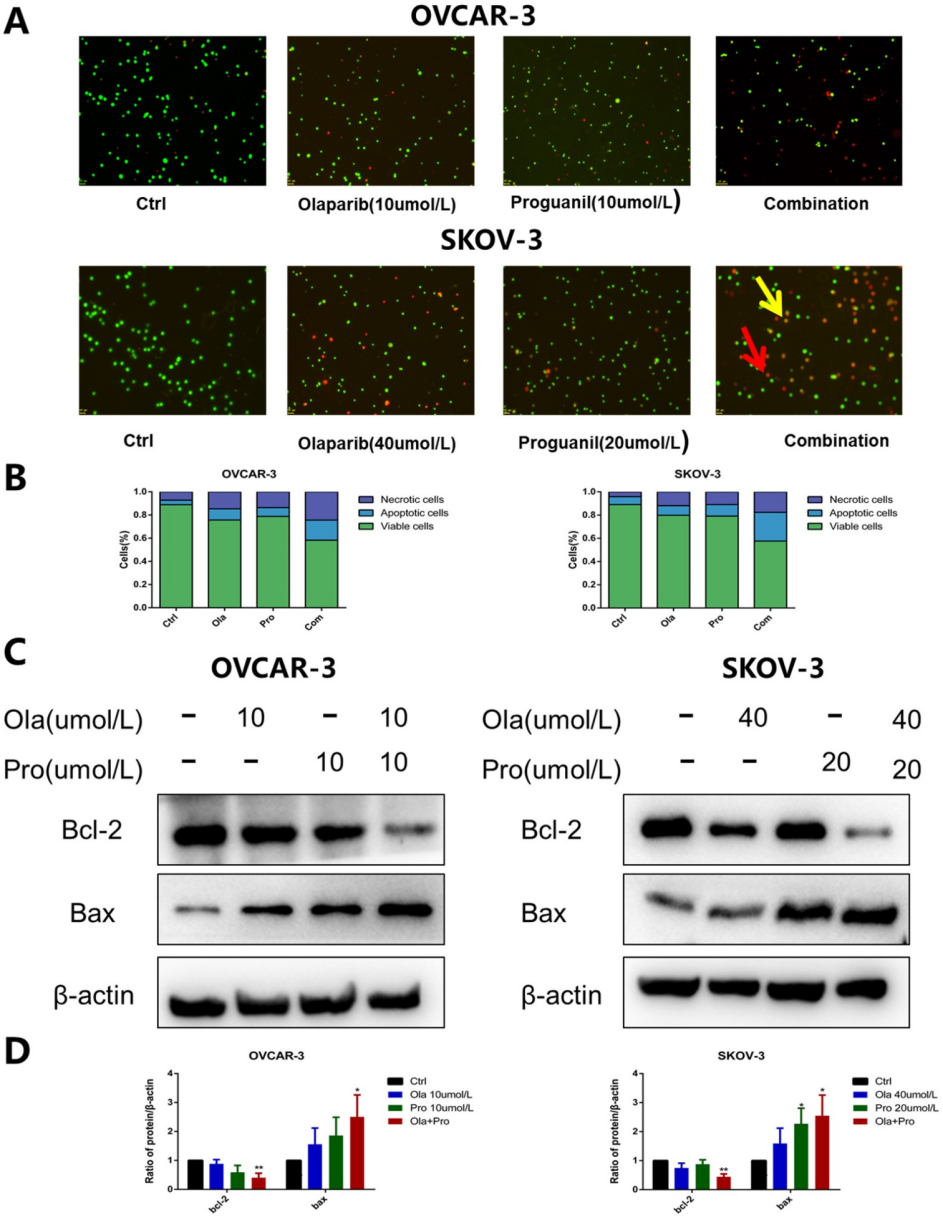
** Olaparib and proguanil synergistically enhance apoptosis of OVCAR-3 and SKOV-3 cells.** (A) AO/EB staining assay in ovarain cells treated with olaparib and/or proguanil was used to examine cell death. The yellow arrows indicate apoptotic cells, which are yellow or orange. And red arrows indicate necrotic cells, which are red. (B) Quantification results were presented. (C) The effects of combination treatment of olaparib and proguanil on the protein expression of Bcl-2 and Bax were analyzed by western blotting. (D) Relative levels of various proteins. (*P < 0.05, **P < 0.01, ***P < 0.001, n = 3.)

To further confirm the effect of the combination of the two drugs on apoptosis, we used the AO/EB staining assay to detect this effect. Unlike flow cytometry, where the principle of detection is to measure the apoptosis-related alterations of cell membrane, AO/EB double staining is to detect the apoptosis-related difference of nucleic acids including the binding of single DNA strand, double DNA strand and RNA. OVCAR-3 cells and SKOV-3 cells were stained with AO/EB and typical pictures were shown (Fig. 3A-B). In the drug combination group, more apoptotic and necrotic cells could be observed than in the single drug-treated.

The Bcl-2 family has an essential role in monitoring the mitochondrial apoptosis pathway 10. Within Bcl-2 family, two most prominent molecules are Bcl-2 and Bax. Bcl-2 is a known anti-apoptotic protein, while Bax is assumed to be a pro-apoptotic protein. It is well known that the intracellular Bcl-2/Bax ratio is a critical factor in determining the susceptibility to apoptosis 11. Our results show that the decrease of Bcl-2 expression and the increase of Bax expression were significantly detected in ovarian cancer cells treated with the combination of olaparib and proguanil (Fig. 3C-D). The decreasing bcl-2/bax ratio represents an increase in susceptibility to apoptosis, as shown in Figure S1, which was consistent with the results stated above. These results suggested that proguanil enhances olaparib-induced apoptosis associated with mitochondria. This observation is consistent with literature which reported that proguanil enhanced the action of atovaquone, causing mitochondrial membrane potential disintegration and leading to mitochondrial dysfunction 12.

### The combination of olaparib and proguanil induces increased DNA damage by reducing homologous recombination repair in ovarian cancer cells

**Figure 4:**
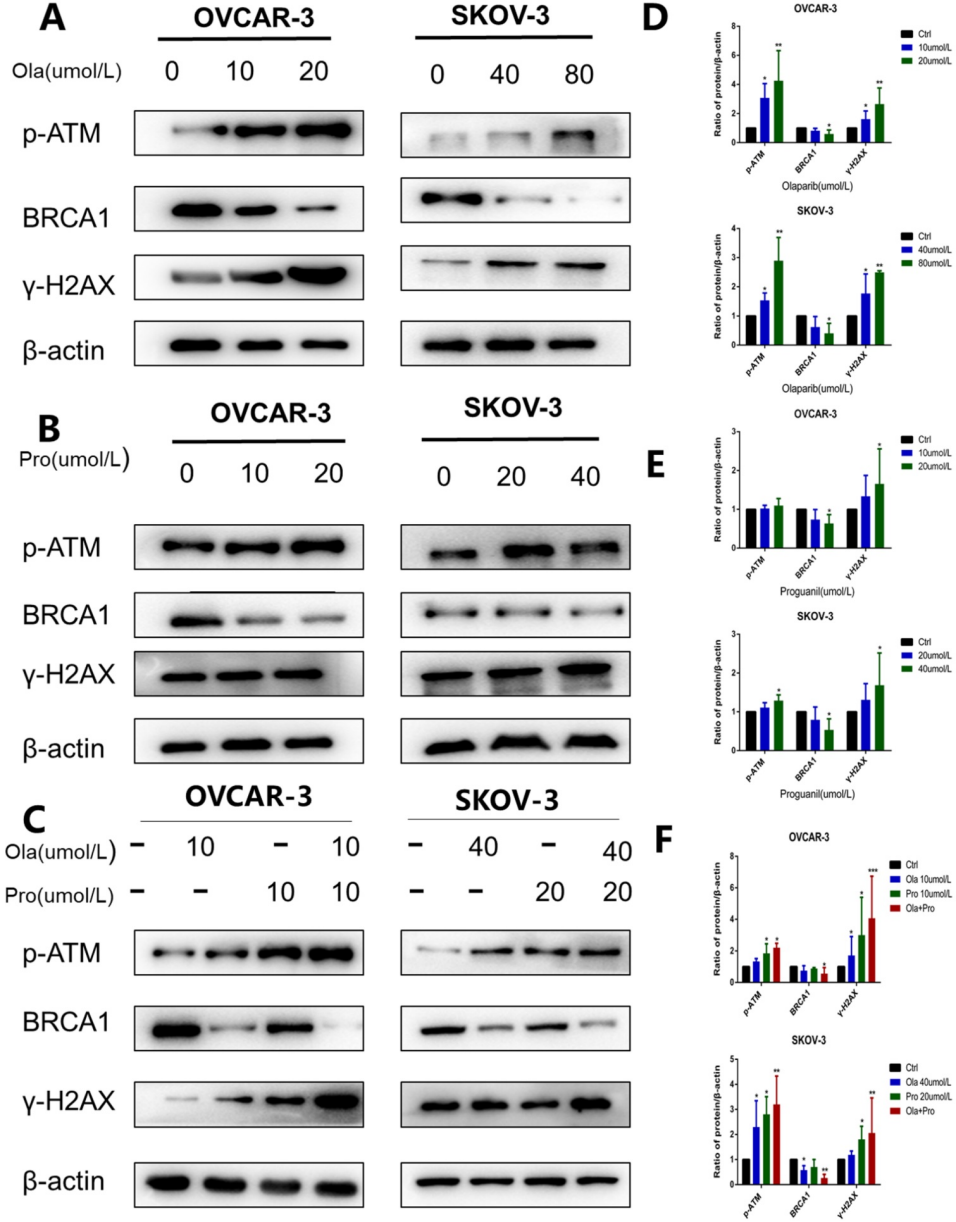
** Effects of olaparib and proguanil alone or in combination on homologous recombination repair signaling.** Western blotting was used to examine the levels of key proteins associated with the HRR pathway after treatment with olaparib and/or proguanil in OVCAR-3 and SKOV-3 cells. (A-C)The protein expression of p-ATM, γ-H2AX, BRCA1 and β-actin were measured, using β-actin as a loading control. (D-F) Relative levels of various proteins. (*P < 0.05, **P < 0.01, ***P < 0.001, n = 3.)

It is well documented that p-ATM and γH2AX are important molecular markers of DNA damage 13, 14, while BRCA1 is an essential protein to support DNA homologous recombination repair 15. To further explore the mechanisms underlying, we used western blotting to examine p-ATM, γH2AX, and BRCA1 protein levels in cells after these different treatments. Consistent with previous observations 16, olaparib alone increased the expression level of p-ATM and γH2AX, and decreased the expression level of BRCA1 (Fig. 4A-B). In contrast, compared with olaparib alone, the effect of proguanil on DNA damage and inhibition of homologous recombination repair was not very strong yet (Fig. 4C-D). Proguanil merely slightly increased the expression levels of p-ATM and γH2AX, and modestly decreased the expression level of BRCA1. However, when two drugs were combined, these effects were much stronger than that of either single drug treatment alone. There was a more remarked increase in p-ATM and γH2AX in the combination group, and a more profound decrease in BRCA1 (Fig. 4E-F). Together, these results demonstrated that proguanil enhanced olaparib-induced DNA damage-related events, and the decrease in BRCA1 expression may reduce the efficiency of DNA homologous recombination repair.

## Discussion

In this study, we demonstrate for the first time that proguanil can sensitize ovarian cancer cells to the small molecular-targeted drug olaparib. Our results suggest that proguanil and olaparib can exert a notable synergistic antitumor effect on ovarian cancer cells. The mechanisms underlying may be related to the increase of olaparib-induced apoptosis and DNA damage, and reduce of the efficiency of DNA HR repair.

Ovarian cancer has a long history of being a highly lethal tumor of the female reproductive system 2. Olaparib, the first-in-class PARP inhibitor for the treatment of ovarian cancer patients, has benefited many ovarian patients 3. One clinical study showed that in patients with platinum-resistant BRCA1/2 mutation ovarian cancer, olaparib as maintenance monotherapy showed a progression-free survival (PFS) of 11.2 months compared to 4.3 months with placebo, a dramatic improvement for treating ovarian cancer patients 17. However, this benefit is very limited clinically due to limitations in the scope of targeted patients, high recurrence rate and drug resistance 18-21. Therefore, it is an urgent need to pursue new treatment options.

Proguanil, originally developed as an anti-malarial drug 5, has been documented with good anti-proliferative effects 7. Compared to metformin, another biguanide compound, the effective anti-tumor concentration of proguanil is considerably low 7, and it has no side effect of lactic acidosis which appears on other biguanide drugs 22. In this study, we for the first time explored the combinational effect of proguanil with olaparib on ovarian cancer. DNA is constantly damaged by environmental attacks and activities of endogenous metabolism 23. DNA damage response is composed of five major complementary pathways that enhance genome integrity and stability 24. HR repair is the main pathway for repairing double-strand breaks 25, and BRCA1 and 2 are important proteins involved in this pathway, of which mutations in these genes predispose to occurrence of tumors including ovarian cancer 26. It has been widely recognized that p-ATM and γH2AX are important molecular markers of DNA damage 27. In the current study, we mainly measured the changes of the expressions of three proteins: p-ATM, γH2AX, and BRCA1. We found that the combination of two drugs increased the expression of p-ATM, γH2AX, and decreased the expression of BRCA1. Thus, it is clear that the combination of two drugs increased DNA damage with decrease of the efficiency of HR repair.

Our study highlights a potentially viable approach for the treatment of ovarian cancer. Wilson et al. suggested that the sensitivity of olaparib can be enhanced by reducing the efficiency of HR repair, therefore targeting this pathway may be a viable antitumor strategy 9. In the present study, our results suggest that proguanil enhances the ability of olaparib to inhibit the proliferation, migration and survival of ovarian cancer cells. These two drugs may exhibit synergistic anticancer activity by mainly decreasing the efficiency of HR repair and increasing olaparib-induced DNA damage and apoptosis. Therefore, the synergistic antitumor effect of olaparib and proguanil promotes a potentially safe and highly effective treatment option for ovarian cancer by combination of these two drugs.

## Supplementary Material

Supplementary figure.

## Abbreviations

MTT, 3-(4,5-dimethylthiazol) 2: 5-diphenyltetrazolium
AO/EB: Acridine Orange-Ethidium Bromide
HR: homologous recombination
DMSO: dimethyl sulfoxide
PBS: fetal bovine serum
γH2AX: phospho-Histone H2AX
p-ATM: phospho-ATM
PFS: progression-free survival
