# Supplementary material for: Proguanil synergistically sensitizes ovarian cancer cells to olaparib by increasing DNA damage and inducing apoptosis
Source: Int J Med Sci. 2022 Jan 1;19(2):233–41. doi: 10.7150/ijms.67027 (PMC8795793; doi:10.7150/ijms.67027)
Supplement: Supplementary file 1 — Supplementary figure. [file ijmsv19p0233s1.pdf]

Figure S1

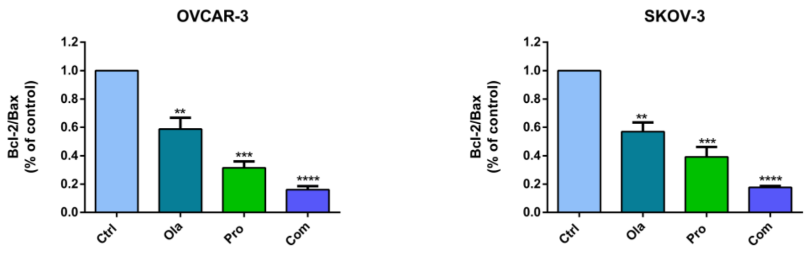

Figure Legend

Figure S1:Olaparib and proguanil synergistically enhance apoptosis of OVCAR-3 and SKOV-3 cells. Western blotting was used to examine the protein expression of Bcl-2 and Bax. The effects of combination treatment of olaparib and proguanil on the ratio of Bcl-2/Bax were measured. (*P < 0.05, **P < 0.01, ***P < 0.001,n = 3.)
